# Supplementary material for: Mechanisms governing the pioneering and redistribution capabilities of the non-classical pioneer PU.1
Source: Nat Commun. 2020 Jan 21;11:402. doi: 10.1038/s41467-019-13960-2 (PMC6972792; doi:10.1038/s41467-019-13960-2)
Supplement: Supplementary file 7 — Source data [file 41467_2019_13960_MOESM7_ESM.zip › Source_Data/FigureS3/FigureS3J_MotifScanOutput/homerResults/motif9.similar.html]

motif9

## Information for motif9

C
A
T
G
A
C
G
T
A
G
C
T
T
A
G
C
T
G
C
A
C
T
G
A
A
C
T
G
A
T
C
G
G
A
C
T
A
G
T
C
C
T
G
A
  
Reverse Opposite:  

G
A
C
T
T
C
A
G
C
T
G
A
T
A
G
C
T
G
A
C
G
A
C
T
A
C
G
T
A
T
C
G
T
C
G
A
T
G
C
A
G
T
A
C
  

|  |  |
| --- | --- |
| p-value: | 1e-133 |
| log p-value: | -3.078e+02 |
| Information Content per bp: | 1.656 |
| Number of Target Sequences with motif | 2601.0 |
| Percentage of Target Sequences with motif | 19.71% |
| Number of Background Sequences with motif | 3986.3 |
| Percentage of Background Sequences with motif | 10.92% |
| Average Position of motif in Targets | 415.6 +/- 374.9bp |
| Average Position of motif in Background | 320.3 +/- 205.4bp |
| Strand Bias (log2 ratio + to - strand density) | 0.0 |
| Multiplicity (# of sites on avg that occur together) | 1.12 |
| Motif File: | file (matrix) reverse opposite |

### Similar de novo motifs found

|  |  |  |  |  |  |  |  |
| --- | --- | --- | --- | --- | --- | --- | --- |
| Rank | Match Score | Redundant Motif | P-value | log P-value | % of Targets | % of Background | Motif file |
| 1 | 0.976 | C A T G A G C T G A C T A T G C G T C A C T G A C T A G A C T G G A T C G A T C T C G A A T G C | 1e-125 | -288.300418 | 21.50% | 12.59% | motif file (matrix) |
| 2 | 0.966 | A C T G A G C T G A C T A T G C C G T A C T G A A C T G A C T G A G C T G A T C C G T A T A G C G C T A | 1e-116 | -267.476165 | 14.49% | 7.43% | motif file (matrix) |
| 3 | 0.897 | C T A G C T G A T A G C G A T C C G A T G A C T C A T G G T C A A T G C G A T C | 1e-106 | -245.713818 | 19.85% | 11.86% | motif file (matrix) |
| 4 | 0.763 | T C A G T C A G A G C T T C A G G T C A A T G C A G T C A C G T G C A T | 1e-105 | -243.376520 | 21.32% | 13.10% | motif file (matrix) |
| 5 | 0.883 | G T A C G T C A C G T A A C T G A C T G A G C T A G T C | 1e-105 | -242.780948 | 35.92% | 25.76% | motif file (matrix) |
| 6 | 0.893 | C G T A C A T G A G C T A G C T T G A C G T C A C T G A C T A G A T C G G A C T A G T C C T G A T A G C G T C A | 1e-84 | -194.572706 | 9.24% | 4.42% | motif file (matrix) |
